# Water (In)Accessibility, Healthcare Delivery, and Patients’ Health Outcomes in Ghana: Perspectives from the Yendi Hospital

**DOI:** 10.3390/nursrep15120418

**Published:** 2025-11-26

**Authors:** Abukari Kwame, Alhassan Siiba, Gervin A. Apatinga, Francis Kwaku Owusu

**Affiliations:** 1 College of Nursing, University of Saskatchewan, Prince Albert Campus, Prince Albert, SK S6V 4E4, Canada; 2 School of Kinesiology & Health Studies, Queens University, Kingston, ON K7L 3N6, Canada; siiba.a@queensu.ca; 3 Department of Community Health Sciences, Cumming School of Medicine, University of Calgary, Calgary, AB T2N 4Z6, Canada; gervin.apatinga@ucalgary.ca; 4 Yendi Municipal Hospital, Yendi P.O. Box 7, Ghana; roukyuk@yahoo.co.uk

**Keywords:** healthcare quality, patient perceptions, patient-provider relationships, water inaccessibility, WASH services, Yendi

## Abstract

**Background**: Access to water, sanitation, and hygiene (WASH) services is internationally recognized as a fundamental human right and an essential determinant of health. Yet, many healthcare facilities in sub-Saharan Africa face persistent WASH deficits, undermining safe and effective care delivery. **Aim**: To explore how water (in)accessibility influences patient healthcare experiences and patient–provider relationships in Yendi Hospital, a major referral facility in northern Ghana. **Methods**: Using a qualitative design, we gathered data from patients (n = 21), caregivers (n = 11), and nurses (n = 11) through in-depth interviews, participant observation, and a focus group to document their lived experiences and perceptions. We transcribed and inductively coded the data for thematic analysis. **Results**: Our key findings reveal that water inaccessibility is not solely an infrastructural issue but also a pervasive challenge with profound implications for care delivery. Patients and caregivers often leave the hospital to bathe at home, resulting in missed ward rounds, delayed reviews, and/or refusal of admission. Nurses described how water inaccessibility disrupted clinical routines and strained relationships with patients and caregivers. These dynamics eroded trust, rapport, and professional morale, while exacerbating inequities in healthcare access and outcomes. **Conclusions**: This study underscores that addressing water challenges in the hospital is imperative not only for infection control but also for fostering equity, patient rights, and institutional resilience. We argue that policy interventions to strengthen WASH systems are urgently required to advance progress toward Sustainable Development Goal 6.

## 1. Introduction

It is well-established that water, sanitation, and hygiene (WASH) services are fundamental determinants of human development, and access to these services is widely recognized as an essential human right [1]. This is explicitly reflected in Goal 6 of the Sustainable Development Goals (SDGs), which seeks to “ensure availability and sustainable management of water and sanitation for all” by 2030 [2] (p. 11). However, as 2030 approaches, significant progress gaps remain, with more than half of the global population lacking adequate, reliable, and clean WASH services [3]. The United Nations’ [3] SDG progress report underscores this concern, revealing that none of the targets under SDG 6 are on track. Specifically, in 2022, an estimated 2.2 billion people lacked access to safe drinking water, while 2 billion had no basic handwashing facilities, amounting to 703 million individuals without basic water services and 653 million without any handwashing facilities [3] (p. 24). These deficits are most acutely experienced in the global south, particularly in sub-Saharan Africa, where structural barriers, systemic inequities, and historically entrenched power dynamics constrain the availability and utilization of WASH services [4,5,6,7,8,9].

The consequences of these deficits extend well beyond basic inconvenience, as inadequate WASH access has profound implications for health and human development [10,11,12]. Limited availability of WASH services undermines maternal and child health [13], compromises the effective functioning of healthcare facilities [14,15], and exacerbates existing social inequities [16,17]. In Gaza, for example, Farajallah and Farajallah [18] found that water inaccessibility adversely affected maternal and child health, with poor access to water increasing the risk of dehydration and malnutrition among pregnant women, leading to poor pregnancy outcomes. For children, unsafe water consumption was associated with heightened incidences of diarrheal diseases and malnutrition, contributing to higher rates of illness and mortality [19,20].

The problem becomes particularly acute in healthcare settings, where reliable access to WASH services is indispensable for safe and effective care delivery. The absence of adequate WASH services in hospitals and clinics constitutes a major public health challenge, exposing healthcare workers to additional occupational risks, especially during public health emergencies such as Ebola and COVID-19, when basic hygiene is critical, but difficult to maintain [18,21,22]. Moreover, inadequate provision of WASH services has far-reaching effects on patient-provider relationships and on perceptions of care quality. In their systematic review, Bouzid et al. [23] found that poor WASH conditions in healthcare facilities were strongly associated with patient dissatisfaction, with some women citing these conditions as a key reason for preferring home births over hospital deliveries. Although the study also noted that healthcare providers’ interpersonal behaviors contributed to dissatisfaction, inadequate WASH services were identified as a central factor in shaping negative experiences of care. Another study by Marquer et al. [24] posited that patients and their caregivers often bear the direct burden of inadequate WASH facilities, being required to bring water themselves for essential practices such as bathing and cleaning, which in turn reinforces inequities in healthcare access [24].

Despite this growing evidence, little is known and written about how water (in)accessibility specifically influences healthcare delivery and outcomes in Ghana. While some recent studies provide important insights into health systems challenges [25,26], they often focus on determinants, such as health insurance and healthcare-seeking behavior [27], occupational health practices among healthcare workers [28], or general inaccessibility issues [29]. Although these studies have made important contributions, they have largely overlooked the critical role of WASH in shaping the quality of healthcare delivery and patient healthcare experiences. Consequently, key insights remain underexplored, which may limit the development of interventions that could effectively address WASH-related challenges in hospital settings and, in turn, hinder or undo progress toward achieving the health-related SDGs. To address this gap, we explore how water (in)accessibility influences healthcare delivery and patient healthcare experiences in northern Ghana. Our findings will enrich and extend the WASH empirical literature while offering critical insights that can guide interventions to mitigate water-related challenges in Ghanaian hospitals and clinical settings, thereby supporting progress toward the realization of SDG 6 and the broader health-related SDGs. The findings from this study may also influence how WASH resources and practices are managed in the Yendi Hospital and other facilities with similar contexts to promote high-quality care and minimize patient-provider conflicts around access to water and hygiene services.

### Geographical Context

**Figure 1:**
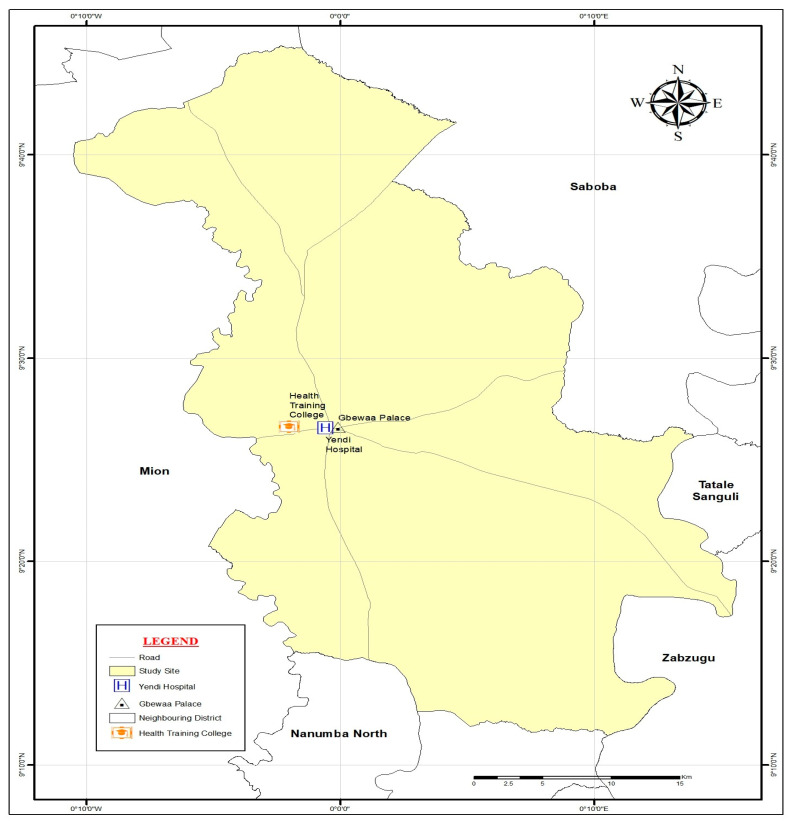
The geographical location of the hospital enrolled in the study.

We conducted the study in Yendi, a major town located in the eastern enclave of Ghana’s Northern Region (see Figure 1). Yendi is the traditional capital of the Dagomba people and the seat of the Yendi Municipal Assembly. According to the 2021 Population and Housing Census, the municipality recorded a population of 154,421 [30]. Strategically situated, Yendi serves as an important hub, linking several districts and towns through economic, cultural, and historical networks.

The Yendi Hospital (located in Figure 1), a key referral facility in the eastern part of the region, was selected because of its central role in healthcare delivery and training. The hospital offers primary care and general medical services, including emergency care, maternal and infant services, reproductive care, surgical services, and serves as a referral centre for other clinics. It serves not only the local population but also supports health training institutions such as the Yendi Health Training College (see Figure 1) and other nearby facilities, which rely on the hospital for clinical practice and professional development. Its strategic importance, therefore, makes it an ideal site for examining how water accessibility intersects with healthcare delivery and patient care experiences.

However, despite its growing population and expanding infrastructure, Yendi remains relatively water insecure. Essentially, in terms of access to basic drinking water services, the municipality ranks 150th out of 261 districts in Ghana [31]. While Yendi performs relatively better than other districts in the Northern Region, it has experienced recurrent annual water crises, particularly during the dry season, for nearly a decade. Data from the Ghana Statistical Service indicate that access to pipe-borne water declined from 21.7% in 2010 to 19.8% in 2021 [32], with significant implications for household health and institutional management.

The Ghana Water and Sewage Company supplies Yendi with water twice weekly, including to the Yendi Hospital. The hospital is also supported by a large overhead water tank and several poly tanks, which are filled when taps are opened. These reserves provide water for patients, caregivers, nurses, and the wider hospital community. However, once the tanks are depleted, access to water becomes highly constrained. This periodic and unpredictable fluctuation in water supply often creates differential experiences among patients and caregivers, posing a structural impediment to consistent care delivery.

Therefore, our choice of the Yendi Hospital for this study was deliberate. Its status as a referral and training centre, coupled with the municipality’s persistent water challenges, presented a unique opportunity to investigate how intermittent water shortages influence patient care and patient–provider relationships.

## 2. Methods

### 2.1. Study Design

This study was part of a doctoral project that explored how institutional practices, power relations, and language ideologies in healthcare settings shape patient rights outcomes in Ghana. An integrated qualitative research design, which combined ethnography, critical discourse studies, and interpretive phenomenology, was employed in this study. These approaches helped us to investigate patient rights within nurse–patient interactions and communication practices at a government hospital in Yendi, Northern Ghana. For this paper, we draw specifically on the ethnographic component to report on the influence of water (in)accessibility on patient healthcare experiences and patient–provider interactions within the hospital. Ethnography is a critical qualitative framework for examining individuals’ everyday experiences and the social norms and practices that shape those experiences in social and organizational settings [33,34]. Guided by this methodological orientation, our analysis interrogates how access to water influences social interactions and patients’ care experiences.

### 2.2. Study Participants and Recruitment

Nurses constitute one of the largest healthcare providers, who interact with patients and caregivers daily to deliver care. As such, understanding the institutional factors that influence their relationships, interactions, and communication practices with patients and patient rights is crucial. Thus, the study participants included nurses, patients, and caregivers, purposively sampled from nine patient units at the Yendi Hospital. Participant recruitment was facilitated by word of mouth and posters. Participation was limited to patients and caregivers 18 years and above who could provide their informed consent and engage in interviews lasting at least 20 min without compromising patients’ health. Patients and caregivers below 18 years and those who could not provide informed consent were excluded. We included nurses with at least three years of practical experience in the hospital. As such, nurses who served less than three years were excluded. This inclusion/exclusion criterion allowed only nurses with in-depth knowledge and experience about the impact of institutional factors on their nursing care and relationships with patients and caregivers to participate in the study. All participants provided voluntary consent by signing and thumb-printing the consent forms or by oral consent, which was audio-recorded by the field researcher. In all, we recruited 11 nurses, 11 caregivers, and 21 patients for the project.

### 2.3. Ethical Approvals

This study was part of a doctoral project, which received ethics approval from the Ghana Health Service Ethics Committee (GHS-ERC:005/11/21), the Behavioural Ethics Committee of the University of Saskatchewan, Canada (Beh-ID: 2690), and the management of the Yendi Hospital in Ghana. Participation in the study was voluntary, and all research participants were respected throughout the project. Participants’ identifying attributes were removed from the reports to promote confidentiality, and serial codes and pseudonyms were used to enhance anonymity. Furthermore, all local protocols governing social interaction in the community and within the hospital were observed since the field researcher is a native and resident of Yendi.

### 2.4. Data Collection

Data were collected using multiple qualitative techniques, including individual interviews, participant observation, a focus group discussion, and informal conversations at nurses’ workstations. The field researcher conducted 11 in-depth interviews with nurses to document their experiences of nurse–patient interactions and communication practices, experiences of patient rights, and barriers to effective care delivery. These interviews, conducted in English and audio-recorded, were guided by a semi-structured interview schedule with open-ended questions. Also, to capture the perspectives of both patients and caregivers, 11 interviews were conducted with caregivers and 17 with patients. While most interviews were conducted in English, four were held in Dagbani with patients and caregivers to accommodate the participants’ preferences. The interview guides were translated into Dagbani by a professional translator proficient in both languages, and the field researcher—a native Dagomba fluent in both English and Dagbani—conducted all interviews. As a native of Yendi and familiar with the hospital environment, the field researcher observed all local community protocols. Each interview guide was piloted with at least three patients, caregivers, and nurses in a different healthcare facility in the same town to ensure that the questions were appropriate and unambiguous. The interviews focused on participants’ experiences of nurse–patient communication, patient rights, and the barriers shaping access to and satisfaction with healthcare [35].

Complementing the interviews, participant observations were undertaken across all nine patient units of the hospital to capture how clinical spaces were organized and to document everyday patterns of nurse–patient interaction, communication practices, and clinical processes. A week was dedicated to each patient unit, after which random observations were conducted in all units throughout the five-month fieldwork. Observations also attended to ideological positioning, the discourses and actions that implicitly or explicitly structure behaviors, experiences, and perspectives in the hospital setting [33,34]. These observations were conducted during nurses’ medication rounds, clinicians’ ward rounds, patient admissions at nurses’ workstations, and informal social interactions within and around patient units. An observation guide facilitated these observations, and consistent and detailed field notes were compiled during and after each session.

Informal conversations with nurses at their workstations provided additional insights into topical issues, including motivation, leadership practices, support for further studies, remuneration, and occupational safety. These conversations were systematically documented in field notes. Additionally, the field researcher held formal meetings with three hospital unit heads to better understand specific managerial processes (e.g., at the patient records unit, lab, and pharmacy) and their influence on care delivery and nurse–patient interactions.

Finally, a focus group was held with four patients awaiting surgery, who had spent several days in the hospital. Their willingness to participate collectively allowed us to capture both individual and shared experiences of nurse–patient relationships, clinical processes, barriers to healthcare access, and patient rights [36]. Bringing these methods together enabled triangulation and illuminated the intersections between personal experiences, institutional structures, and broader social norms. Such an integrated approach is particularly critical in ethnography, which emphasizes the connection between people’s everyday experiences and the wider social and institutional arrangements that shape them. In our opinion, combining these methods generated richer and more reliable insights into how water access mediates patient–provider interactions and healthcare delivery processes in the context of Yendi Hospital.

### 2.5. Data Analysis

Data analysis was iterative, with Braun and Clarke’s [37,38] reflexive thematic analysis providing the overarching framework for analysis. To facilitate this, interviews were transcribed progressively; initial interviews were transcribed before subsequent ones were conducted, allowing the field researcher to identify and further probe emerging issues during later data collection. All interviews conducted in English were transcribed verbatim, while those in Dagbani were translated into English during transcription by the field researcher. To minimize the loss of meaning, key Dagbani concepts and phrases were retained in their original form.

Transcripts from patients, nurses, and caregivers were separately coded manually and inductively to capture the unique experiences of each group. Field notes were subjected to the same process. Each transcript was read multiple times to ensure deep familiarization before inductive coding was carried out. Codes were initially broad and captured all potential barriers to effective patient–provider relationships and care delivery (e.g., cost of care, language and communication, water access, health beliefs, long waiting times, lack of essential medicines, staff shortages, and limited medical/nursing supplies).

From this comprehensive coding process, barriers specifically related to water (in)accessibility were identified and grouped according to whether they arose from the experiences of patients, nurses, or caregivers. These codes were then categorized into two major domains: those that influenced patient healthcare experiences (e.g., missed medical reviews, refused hospital admission, risks of infections, cost of buying water, etc.) and those that affected patient–provider relationships (e.g., nurses’ attitudes, desire to serve in the hospital, ward hygiene, etc.). Following Braun and Clarke’s [37,38] reflexive thematic approach, the analysis ultimately yielded three interrelated themes with sub-themes: (1) perceptions of water (in)accessibility, (2) impacts on patient care, and (3) effects on patient–provider relationships. Verbatim excerpts from participants were incorporated to substantiate the findings. To ensure confidentiality and protect participants’ identities, all interviewees, focus group participants, and caregivers were assigned unique identification codes.

### 2.6. Rigour in Data Analysis

Several processes and activities were performed to ensure the findings are credible and trustworthy [39]. Firstly, a multi-data collection approach was undertaken to gather in-depth information to understand the complex factors determining nurse-patient interactions, communication, relationships, patient rights, barriers to care delivery/access, and care experiences. These data sources complemented each other to provide a broad picture of the research problem. Secondly, many participants (all nurses and a few patients/caregivers) reviewed their interview transcripts and provided feedback, including clarifying some sentences, terminology, or adding a few more ideas. This feedback enriched the data. Thirdly, preliminary findings of the study were shared with the hospital community and presented at doctoral student seminars. Feedback from these preliminary findings encouraged further reflection and interpretation of the data, leading to high-quality study reports. Lastly, the field researcher also engaged with their doctoral research committee throughout the project to gain expert peer reviews and guidance. These processes and activities ensured that the study outcome was credible, dependable, and trustworthy. Community members and healthcare providers could relate to the project findings, as it confirmed their lived experiences of delivering or accessing care from the hospital.

## 3. Results

### 3.1. Participant Demographics

A total of 43 individuals participated in the study, of whom 27 were females. The sample included 21 patients, 16 of whom were females, with ages ranging from 18 to 60 years (M = 26). The majority of patients were Dagomba. Patients’ average length of hospital stay was three days, with the longest stay extending to two weeks. Educational attainment varied, though most patients had completed secondary school. The sample also comprised 11 caregivers (7 females), aged 19 to 45 years (M = 32). Most caregivers reported basic educational qualifications and were predominantly Dagomba. Notably, caregivers’ length of stay at the hospital closely mirrored that of the patients, which shaped their perspectives on water accessibility in the facility. In addition, nurses were 11, four of whom were females, aged 26 to 40 years (M = 33). Most nurses were married, Dagomba, and had an average of six years of professional practice in the hospital. Participant demographic characteristics are summarized in Table 1.

### 3.2. Theme 1: Perceptions of Water Accessibility in the Hospital

Participants expressed mixed perceptions of water accessibility in the hospital, reflecting their varied experiences. While some patients and caregivers considered water access manageable, others described it as a persistent challenge. The data revealed this diversity of views, as six out of 11 caregivers and nine out of 21 patients reported that water access was not a challenge, while four caregivers and nine patients said it was. The remaining four patients and one caregiver did not know or respond. Table 2 provides excerpts of participants’ responses.

Interview narratives further illustrate these divergent positions. Some participants expressed confidence in the hospital’s supply: “*I don’t really struggle for water here; it’s usually available*” (Patient 8, female) and “*It’s not a challenge. We have not had any challenges with water*” (Patient 16, male). Others, however, highlighted difficulties: “*Sometimes you can search all over and still not get water in the tanks*” (Caregiver 4, female).

Interestingly, caregivers who initially said water was not a challenge often used down-toners or qualifiers (e.g., well, not big, not severe, and not very, etc.) in their responses, revealing underlying difficulties: “*It’s not too serious; we usually get some water, but not always when we need it*” (Caregiver 6, male), “*It’s not a big challenge to get water here*” (Caregiver 9, female) and “*It’s minimal; the challenge of getting water is not severe*” (Caregiver 3, female). This finding resonates with patients’ remarks such as, “*It’s okay, not really a big issue, but occasionally it’s hard to find water*” (Patient 1, female) and “*Access to water is not a big problem*” (Patient 6, female).

Participant observations validated these mixed accounts. On some days, caregivers were seen drawing water with ease from poly tanks, while on other days, they struggled and queued to fetch limited quantities from the tanks. These observations mirrored the confidence and concerns expressed during interviews, confirming that water availability fluctuated and that perceptions of accessibility depended on when and how participants experienced these fluctuations.

### 3.3. Theme 2: Impact on Patients’ Care

This theme describes how water (in)accessibility shaped patients’ health, personal hygiene practices, and overall experiences of care in the hospital. Evidence from interviews, focus groups, participant observations, and nurses’ narratives consistently validated one another across four subthemes.

#### 3.3.1. Sub-Theme 1: Missed Medical Reviews

Patients reported missing scheduled reviews because they had to leave the ward to bathe at home. One explained: “*I told the nurse I needed to go home and wash, but by the time I returned, the doctor had already checked other patients. The nurse was upset and said it seemed I was well enough to be discharged*” (Patient 15, male). Another added: “*I wanted to bathe in the afternoon, but the nurse advised me to wait until the doctor had finished rounds, then I could leave and return before evening medication*” (Patient 10, male).

Participant observations confirmed these reports. In the pediatric unit, a field note recorded: “*A young mother missed her child’s medical review because she had gone home to bathe. Another child’s surgical assessment was delayed for the same reason. At first, I was surprised, but later learned this behavior was due to unstable water supply in the hospital*” (Field note, 28 February 2022).

Together, interviews and observations show that both adult and pediatric patients regularly missed critical/medical reviews because they left the hospital to manage hygiene needs at home.

#### 3.3.2. Sub-Theme 2: Influence on Patient Admission

Nurses’ interview data revealed that water shortages discouraged patients from accepting hospital admissions. As one noted: “*Some patients refuse to stay even when they need admission. They say the bathrooms and water supply are not good, so they prefer going home*” (Nurse 3, male). Another nurse explained: “*Patients sometimes complain about the environment. Because of the water challenges, they choose not to be admitted*” (Nurse 4, male).

This pattern was consistent with some patients’ interviews, where reluctance to remain in the hospital was linked to challenges with water and sanitation facilities. Also, observation captured some of these challenges as noted here: “*Access to water and patient care in the hospital ward seems to be a persistent issue. Can the lack of access to water and sanitation resources in the wards affect patient behaviours and care experiences? I have seen caregivers bring hot water from home for their patients in the labour and maternity wards*” (Field notes, 14 January 2022). Thus, observations of patient turnover validated these accounts, showing that water inaccessibility influenced decisions about hospitalization.

#### 3.3.3. Sub-Theme 3: Personal Hygiene Needs

Caregivers and patients described daily struggles to maintain hygiene due to an unreliable water supply. One caregiver observed: “*Look at that tank—it’s empty. We go around checking which one has water. Sometimes you only get one bucket, and it’s not enough if you have clothes to wash*” (Caregiver 11, female). Patients echoed similar frustrations: “*Access to water is a challenge. Toilets often don’t have water, and washing clothes is very hard for caregivers*” (Patient 17, male).

Focus group participants reinforced this theme, emphasizing poor hygiene conditions in the wards and washrooms. “*The hygiene needs a lot of improvement. Sometimes, after using the toilet, you don’t feel like eating*” (Focus Group Participant 4, female). Another added: “*Before I go in, I wear a nose mask because the place is so unpleasant*” (Focus Group Participant 3, female).

Patient observations further confirmed that caregivers often left wards searching for water, while cleaners sometimes restricted toilet use to maintain hygiene: “*Patients complained that cleaners asked them to urinate outside after early-morning cleaning*” (Patient 13, female).

Together, these accounts reveal how an inadequate water supply directly affected patients’ and caregivers’ personal hygiene and their perceptions of care quality.

#### 3.3.4. Sub-Theme 4: Increased Costs of Care

Several participants highlighted the financial burden of purchasing water in the hospital. A caregiver explained: “*When we need hot water, we have to buy it. Early in the morning, someone sells it here*” (Caregiver 7, male). Also, a patient in the focus group elaborated: “*One container of hot water costs 50 pesewas ($0.08), and usually you need two. If you have washing to do, it can cost more than one cedi*” (Focus Group Participant 4, female).

This added cost was significant for already burdened families. When asked what message they wanted to give hospital leaders, one caregiver responded: “*They should make water very accessible in the wards; it will make life better for patients and caregivers*” (Caregiver 7, male). Similarly, a patient stressed: “*Water is the main problem. Each ward should have water to make life comfortable*” (Patient 14, female). These reported experiences suggested an added burden to the cost of care for patients and caregivers.

### 3.4. Theme 3: Nursing Care and Patient–Provider Relationship Quality

Water (in)accessibility strongly influenced patient–provider interactions, nursing care practices, and perceptions of relational quality. The data from interviews, participant observation, and informal conversations consistently validated one another across six sub-themes.

#### 3.4.1. Sub-Theme 1: Provider Frustration with Patients

Patients sometimes missed ward rounds and medications because they had to leave the hospital to bathe at home, which generated frustration among healthcare providers. One patient recounted: “*When I returned after 10:00 am, the doctor had already checked other patients. The nurse was unhappy and said to me, ‘It seems you have recovered because you went home and didn’t come back, even when the doctor was here. I think they should discharge you now.’*” (Patient 15, male).

Participant observation confirmed similar tensions in the Children’s Ward: “*The charge nurse was unhappy with two mothers who missed their children’s medical reviews because they had gone home to bathe. One child was scheduled for surgery while the other was on admission for treatment*” (Field note, 28 February 2022). These examples illustrate how water scarcity directly fueled nurses’ frustration toward patients and caregivers.

#### 3.4.2. Sub-Theme 2: Missed Opportunities for Rapport

Participant observation revealed missed opportunities for building trust between caregivers and nurses. A field note detailed: “*Today, while I was conducting participant observation in a patient ward, a caregiver came to beg for water from the nurses*. *However, she was refused and asked to go to the maternity ward. The nurses told the woman that there was a pipe there or that she could go to the kitchen side. Since this caregiver didn’t need much water, I thought it was an opportunity for the nurses to build a nurse–caregiver relationship with the woman*” (Field note, 17 January 2022).

Such observed moments highlight how water inaccessibility created small but critical fractures in patient–caregiver–nurse relationships.

#### 3.4.3. Sub-Theme 3: Conflicts over Responsibility

Conversations with nurses revealed instances of conflict with caregivers regarding water responsibilities. During a discussion at the nurses’ workstation, one nurse recounted: “*Once, a caregiver poured water out of the ward through the window. When I asked him why, he said it was my duty to get a container in the ward so that they could pour dirty water into it since there was no water in the ward*” (Field note, 31 January 2022). This illustrates how caregivers’ frustrations about water shortages could escalate into interpersonal conflict and contestations over responsibility for managing water and waste.

#### 3.4.4. Sub-Theme 4: Nurses’ Personal Burden

Nurses themselves experienced the consequences of water scarcity, particularly those living in hospital-provided accommodation facilities. A field note described: “*During a conversation today at the nurses’ station, they indicated that the hospital-provided accommodation facility for nurses was in poor condition and that sometimes, nurses living in those housing units walk a distance to fetch water for themselves*” (Field note, 8 January 2022). Similarly, a nurse was talking about the challenges they face at the OPD and stated that “*there are times when there is no stable water flow, which impacts their work, because they have to fetch water from outside for use in the sluice room*” (Field note, 6 February 2022). This highlights how institutional water challenges not only affected patient care experiences but also shaped the daily lives and working conditions of nurses, compounding stress and fatigue.

#### 3.4.5. Sub-Theme 5: Concerns About Infection Control

Nurses repeatedly expressed concerns about infection prevention under conditions of water scarcity. A field note captured: “*Today, the matron and the hospital administrator came to the labour ward during their administrative rounds. The nurses told them there was a high rate of infections in the ward, and that immediate attention was needed to unclog the sink and provide additional containers for water*” (Field note, 17 January 2022).

Similarly, in interviews, one nurse noted: “*Here (in this unit), our toilet is not good for use. We even had to call someone to come and work on it. Also, there is a water challenge*” (Nurse 5, female). Nurses in the labour and maternity wards reported having to organize containers in the ward to store water during shortages, further illustrating how the absence of a reliable supply heightened fears of hospital-acquired infections.

#### 3.4.6. Sub-Theme 6: Impact on Career Aspirations

Water scarcity also influenced future workforce considerations. A student nurse, admitted as a patient, described:

*R: As a patient, have you experienced any challenges in this hospital? Let’s start with water. Is access to water a challenge for you?*

*P9: It is. That is very difficult.*

*R: Meaning that it is a challenge for you?*

*P9: Yes.*

*R: What about hygiene in the ward?*

*P9: It’s ok.*

*R: If you had the chance to talk to the hospital management about things you want them to change or improve, what would you tell them?*

*P9: First, it will be the fans, because the room is very hot. That would have been the first thing I would mention, and then water; it’s important.*

*R: Would you like to work in this hospital?*

*P9: Yes, if only things were changed (laughs).*

*R: Please explain what that means.*

*P9: Mm, if things like the water problem and others get changed. Because right now I am seeing problems in the hospital, I won’t be happy working here. I see that patients and nurses are already going through challenges, including water problems.”*

This account illustrates how water inaccessibility not only influenced patient satisfaction but also discouraged young professionals from envisioning a future career in the hospital.

## 4. Discussion

This study explored water accessibility issues at the Yendi Hospital by examining nurses, patients, and caregivers’ perspectives. The findings showed interesting dynamics, especially about perceptions of water accessibility at the hospital. Although many participants, especially caregivers and patients, reported not facing significant water challenges in the hospitals, their narratives actually revealed that these participants underrated their experiences, as many quantifying, down-toners, and discourse markers were used to introduce their responses. Incidents of water inaccessibility have been reported in other healthcare facilities in the region, including the Tamale Teaching Hospital, exposing underlying healthcare infrastructural deficits and accountability challenges [40]. Similarly, Kwakye [41] observed that in healthcare facilities across the Ga West Municipality of the Greater Accra region, many patients reported low satisfaction with water, sanitation, and hygiene services in those facilities.

Participants in this study reported missed medical reviews, increased costs of care, and patients refusing admission to the hospital due to a lack of stable water flow and limited sanitation and hygiene services. These findings confirm previous research results both in Ghana and elsewhere. For instance, a systematic review of global evidence shows that poor WASH deters some patients from seeking care or returning to the facility, reducing preventive and follow-up visits and increasing late presentations [23].

In addition, our study found that unstable water supply in the hospital affected patients’ hygiene needs and increased perceived risk of infections, corroborating findings in the literature that when water is intermittent or absent, handwashing compliance drops and facility infection control practices become compromised, increasing risk of healthcare-associated infections [42,43]. Similarly, multisite WASH audits in Kenya showed that wards with limited water have more gaps in handwashing stations, personal protective equipment reprocessing, and environmental cleaning, which directly contribute to healthcare-associated infection (HAI) risk [43]. Again, regarding surgical safety, Chawla et al. [44] observed that in low and middle-income countries, water shortages constrain instrument cleaning and perioperative hygiene, which reduces the capacity to deliver safe surgical care and raises postoperative infection risks.

Nurses, especially in the maternity and labour units, lamented the impact of water inaccessibility on their care delivery practices and patient safety, confirming research findings in Ghana and other jurisdictions that indicate the critical role stable water supply plays in maternal, newborn, and postnatal outcomes [25,45]. Inadequate water and sanitation in maternity units are associated with poorer hygiene during delivery and postpartum care, resulting in low WASH Facility Improvement Tool (WASH-FIT) scores with maternal infection risks and lower perceived quality of intrapartum care [23,45]. Moreover, some patients complained about sanitation in patient washrooms in the unit, confirming Ashinyo et al.’s [25] finding that postnatal women report concerns about facility WASH (privacy, cleanliness), which was linked to lower satisfaction and reduced likelihood of returning for follow-up, thereby affecting continuity of care and early detection of complications.

Regarding the impact of water inaccessibility on patient-provider relationships, this study found that water accessibility challenges caused patient-provider conflicts, as some patients missed clinical reviews. The WASH literature shows that visible hygiene failures erode patient-provider trust [25]. Other studies have shown that patients who observe poor hand hygiene, unclean wards, or visibly compromised sanitation report lower confidence in clinical competence and safety, which erodes the basic trust needed for effective therapeutic relationships [25,43]. Moreover, postnatal mothers reporting water and privacy concerns stated they felt less safe and less likely to follow postpartum instructions or return for follow-up care [25]. Similarly, studies have shown that lack of functioning toilets, inadequate water for washing, and poor cleaning reduce patients’ sense of dignity, particularly for women in maternity units, leading to shame, reluctance to discuss intimate concerns, and strained interactions with clinicians [23,45,46].

Furthermore, our study found that water accessibility affected patient-provider communication, leading to missed opportunities for rapport building. Findings from research conducted in Asokore Mampong in the Ashanti region of Ghana suggest that patients who are worried about hygiene or privacy may fail to communicate their symptoms or social needs (e.g., substance use, incontinence) to healthcare providers for fear of embarrassment, which impedes accurate diagnosis and effective counselling [26]. Provider defensiveness and time pressures can also affect patient-provider relationships due to water shortages. Studies have found that when water shortages force healthcare providers into improvised care, they may appear hurried or defensive, and when patients interpret these behaviours as a lack of caring, it weakens open and therapeutic communication [42].

Lastly, our study found that some nurses had to search for water to shower before going to work. This situation affected some nursing students’ desire to serve in the facility, confirming findings in the literature, especially concerning provider workload and missed tasks due to water inaccessibility [42,43]. For instance, facility audits and staff interviews in Kenya document that staff must allocate time to secure water (fetching, improvising), which reduces time for clinical tasks and case management, contributing to care delays and procedural shortcuts that harm care outcomes [42]. Similarly, water shortages often co-occur with limited WASH supplies (soap, disinfectant), amplifying risks and constraining implementation of clinical guidelines [43].

### Actionables for Healthcare Practice, Management, and Policy

Our study explored how water (in)accessibility influences healthcare delivery and patient healthcare experiences in northern Ghana. However, the qualitative design and relatively small sample size restrict the extent to which the results can be generalized. Also, the possibility of social desirability bias cannot be ruled out, as some participants may have overstated certain issues in their responses. Despite these limitations, this study represents one of the few empirical inquiries in Ghana to specifically explore the impact of water inaccessibility on healthcare delivery and patient healthcare experiences in the Northern Region of Ghana. Our findings have implications for care delivery, management, and policy in Ghana and elsewhere with similar situational factors. Invest in reliable water infrastructure, integrate WASH into quality metrics, and include patient voices in WASH improvement planning to restore dignity and relationship quality.

First, investments in water infrastructure at the Yendi Hospital will produce gains. The participants’ experiences demonstrate that more resources for water supply and storage are needed. Studies elsewhere have shown that healthcare facilities that implement reliable water systems (on-site storage, piped supply, contingency plans) plus WASH-FIT–guided improvements improved patient satisfaction, measurable reductions in hygiene gaps, and better maternal outcomes [43,45].

Secondly, integrating WASH audits into the facility quality metrics will help prioritize investments and track outcome-relevant service improvements. This process will determine areas of severe water challenges and their impact on patient-provider relationships and care experiences [23,45].

Patient and caregivers’ concerns over unstable water supply and ward hygiene showed that patient-provider conflicts do emerge. Ensure basic contingency supplies (e.g., soap, bottled water, and secure portable handwashing stations), visible cleaning routines, and clear signage. These pragmatic measures will reduce visible hygiene failures in the facility and preserve patient confidence [43].

Lastly, healthcare managers must train providers to implement therapeutic communication strategies (e.g., transparent explanation, apology, plan) that acknowledge infrastructure limits. Genuinely understanding patients’ and caregivers’ concerns about WASH can preserve trust even when resources are constrained.

## 5. Conclusions

Water, sanitation, and hygiene (WASH) services are fundamental determinants of human development, and access to these services is internationally recognized as an essential human right. Yet, a significant proportion of the global population still lacks access, with the majority of those affected residing in sub-Saharan Africa. The implications for health and well-being are profound, particularly in healthcare settings, where reliable WASH services are indispensable for safe and effective care delivery. This study was conducted at the Yendi Hospital in northern Ghana, a key referral facility in the eastern enclave of the region that has faced recurrent annual water crises. The study examined how water inaccessibility influences healthcare delivery and patient healthcare experiences. The findings reveal that water inaccessibility is not just an infrastructural issue but a pervasive challenge affecting care delivery. Participants’ perceptions ranged from ease of access to persistent difficulty, with many using language that revealed hidden struggles.

Water inaccessibility had tangible effects on patient care experiences. Patients and caregivers often left the hospital to bathe at home, which led to missed ward rounds and delayed medical reviews, while others refused admission altogether due to poor facilities. Nursing care and patient–provider relationships were also disrupted. Nurses frequently expressed frustration when patients missed reviews, while caregivers’ requests for water sometimes led to tensions or missed opportunities to build rapport.

Together, these findings underscore that water inaccessibility undermines not only the quality of care but also the social dynamics of care delivery, including trust, rapport, and professional morale. In the context of the Sustainable Development Goals, particularly Goal 6, addressing water challenges in Yendi Hospital and in similar facilities across resource-constrained settings is not optional but imperative. Water access is not only a matter of infrastructure, but also of equity, patient rights, and institutional resilience.

This study provides critical insights into water provision and access in a low-income healthcare setting and highlights the urgent need for policy attention. Governments, policymakers, and non-governmental organizations must prioritize strengthening WASH systems in Yendi Hospital and other facilities in Ghana facing similar challenges. Doing so will enhance patient care experiences, reduce perceived risks of infection, improve patient–provider relationships, and support a more motivated and sustainable health workforce. Ultimately, such measures will accelerate progress toward achieving SDG 6 and advance health equity in northern Ghana. Lastly, we call for comparative research on this topic within and across different regions of Ghana, especially within teaching and regional hospitals.

## Figures and Tables

**Table 1:** Participants’ demographic data.

Characteristics	Patients	Caregivers	Nurses
Sample size	21	11	11
Sex			
Male	5	4	7
Female	16	7	4
Marital status			
Married	9	8	9
Single	12	3	2
Age (in years)			
Range	18–60	19–45	26–40
Mean age	26	32	33
Ethnicity			
Dagomba	13	8	8
Ewe	2	2	0
Konkomba	2	0	0
Others	4	1	3
Level of Education			
None	3	2	0
Basic	2	6	0
Secondary	10	1	0
Tertiary	6	2	11
Occupation			
Nursing	0	0	11
Student	7	1	0
Trading	4	2	0
Farming	3	6	0
Teaching	3	1	0
Other	4	1	0

**Table 2:** Participants’ perceptions of access to water in the Yendi Hospital.

Patient Responses		Caregiver Responses	
No	Yes	No	Yes
“No, not difficult” (Patient 8, female) “No, access to water is ok” (Patient 12, female) “No, it’s not difficult” (Patient 13, female)	“Yes, it’s challenging” (Patient 2, female) “Hmm, here, water is difficult to get” (Patient 4, female) “Yes, it’s a challenge” (Patient 7, male)	“No water challenges” (Caregiver 5, male) “No, I don’t face water problems” (Caregiver 8, female),	“Yes, it’s an issue” (Caregiver 4, female) “Yes, accessing water is a challenge” (Caregiver 11, female)

## Data Availability

The data used in this manuscript are part of a doctoral research project and contain sensitive or confidential participant information. The data can be made available by the corresponding author upon reasonable request.

## Public Involvement Statement

Patients, caregivers (patient relatives), and nurses were engaged in this study as research participants who provided data through interviews, a focus group, and informal conversations during participant observation sessions. A youth pressure group (The Concern Citizens of Yendi—CCY), although not formally engaged in the study, provided the field researcher with insights on issues of alleged medical diligence. A copy of the project report was provided to CCY after the study was completed.

## Guidelines and Standards Statement

The authors are experienced qualitative researchers, and so they did not follow any qualitative research reporting guidelines while drafting this manuscript.
